# Unpacking Firearm Access and Firearm Violence Exposure Among American Indian or Alaskan Native and Black Adults

**DOI:** 10.1001/jamanetworkopen.2024.0020

**Published:** 2024-03-04

**Authors:** Amelia C. Mueller-Williams, Lara N. Coughlin, Jason E. Goldstick

**Affiliations:** Addiction Center, Department of Psychiatry, University of Michigan, Ann Arbor; Addiction Center, Department of Psychiatry, University of Michigan, Ann Arbor; Michigan Innovations in Addiction Care Through Research and Education, University of Michigan, Ann Arbor; Injury Prevention Center, University of Michigan, Ann Arbor; Injury Prevention Center, University of Michigan, Ann Arbor; Department of Emergency Medicine, University of Michigan, Ann Arbor; Institute for Firearm Injury Prevention, University of Michigan, Ann Arbor

Firearm violence poses a significant and increasing threat to well-being in the US. Per-capita firearm mortality rates increased by nearly 40% from 2014 to 2021, with especially large growth in the last 2 years of official data (2020–2021).^1^ Black and American Indian or Alaska Native US residents have seen the largest per-capita increases in firearm mortality during that time (2014–2021) among all races in the US,^1^ which is emblematic of distinct patterns of racial inequities in firearm death. At the same time, firearm injury epidemiology also differs substantially between American Indian or Alaska Native individuals and Black individuals. Among American Indian or Alaska Native individuals, roughly half of all firearm deaths are suicides, while homicides represented approximately 80% of firearm deaths among Black US residents in 2021.^1^ Research characterizing firearm access and firearm violence exposure among American Indian or Alaska Native and Black adults is sorely needed, but the implications for prevention likely differ between the 2 groups.

In their descriptive study, Anestis et al^2^ contribute much-needed information on the prevalence of firearm access, related behaviors, and exposures to types of firearm violence among a national survey sample of American Indian or Alaska Native and Black adults in the US. Findings from the survey show that home firearm access was common for both American Indian or Alaska Native (45.5%) and Black (30.4%) participants.^2^ Firearm access was most common among rural-dwelling males, and handguns were the most reported gun type. The study found an all-or-nothing pattern for most firearm storage practices among both groups, with more than 75% responding either “always” or “never” to questions on keeping at least 1 firearm loaded and storing firearms using a locking device (eg, cable lock).^2^ However, it was more common to report always keeping firearms in a locked location compared with using a locking device,^2^ suggesting possible avenues for improved secure storage uptake. Regularly carrying a firearm outside the home was reported by a similar minority of American Indian or Alaska Native (18.9%) and Black (15.2%) participants with firearm access, with self-protection or protection of others as the most cited reason.^2^ Interestingly, approximately 15% of both groups reported lack of faith in the police as a reason for carrying a gun outside the home.^2^

Firearm ownership, storage, and carrying choices need to be contextualized within rates of firearm violence exposure, which are elevated in both populations. The lifetime prevalence of being threatened with a firearm was 30.2% for American Indian or Alaska Native adults and 21.7% for Black adults.^2^ Environmental exposure to firearms was also concerning; 27.9% of American Indian or Alaska Native respondents and 38.2% of Black respondents reported having heard or seen a shooting in their neighborhood.^2^ These findings among Black adults are consistent with previous studies showing higher rates of firearm exposure in Black and Hispanic populations relative to their White counterparts^3^ and provide needed evidence on exposure specific to American Indian or Alaska Native adults—an important contribution of this work. Findings from this study also provide additional context for recent work suggesting cumulative firearm violence exposure is associated with poorer health among American Indian or Alaska Native and Black US residents.^4^ Given the numerous health-related sequelae associated with violence exposure,^4,5^ the results from the study by Anestis et al^2^ should be situated within the health disparities that affect American Indian or Alaska Native and Black communities.

The high prevalence of firearm violence exposure reported in these 2 groups is reflective of broader health disparities and differential exposure to adverse social determinants of health that are the product of ongoing structural inequities. These factors propagate cyclically to influence not only violence but also risk factors for violence. For example, American Indian or Alaska Native and Black children experience the highest and second highest rates, respectively, of adverse childhood experiences, which are linked to poverty^6^ and poor health outcomes associated with interpersonal violence exposure.^5^ Structural racism is also associated with substance use, which is both a consequence of and a risk factor for violence exposure.^5^ Alcohol use, in particular, can enhance the proximal risk for firearm violence, with heavy alcohol use at the time of the incident implicated in 20% to 30% of firearm injuries, suicides, and homicides.^7^ At the same time, any approach to preventing firearm injury in these communities should meaningfully consider the key similarities—and differences—in how American Indian or Alaska Native and Black US residents experience structural racism (eg, historical trauma, racial trauma)^8^ and the dynamics of how they are affected by firearm violence.

Given the interconnectedness of health disparities and their common causes in these groups, interventions addressing social determinants of health could be a high leverage point for alleviating not only disparities in firearm-related outcomes but also other outcomes, such as substance use–related harms. At the community level, interventions that target social determinants of health could efficiently address multiple health outcomes while avoiding blaming individuals and communities for structurally constructed issues. Individual- and clinical-level interventions are also needed to mitigate the risk of exposure to firearm violence. Anestis et al^2^ emphasize potential areas for tailored interventions that may be more acceptable in these populations, such as firearm safe storage programs providing gun safes vs cable locks.

Initiatives addressing firearm-related risks should be culturally appropriate and respectful of the complicated etiology of firearm violence that is unique to American Indian or Alaska Native (eg, more driven by suicides) and Black (eg, dominated by homicides) communities. While social determinants of health may impact both groups in similar ways to perpetuate cycles of risk exposure, these populations’ specific experiences with structural racism and intergenerational trauma can be very different. Accordingly, effective strategies to reduce firearm violence exposure among American Indian or Alaska Native and Black US residents should emphasize community-voiced needs while also attending to observed epidemiological trends in firearm mortality and antecedent risk exposure. Research is especially needed on types of firearm access and behaviors that are risk-enhancing and the nuanced interaction between firearms and substance use—especially alcohol.

Firearm violence is a multilevel problem in the US, which necessitates a multilevel solution. Descriptive epidemiological studies on firearm exposure and related behaviors, such as this study by Anestis et al,^2^ provide critical foundations on which to build the multilevel framework needed to reduce firearm injury. Integrated approaches, including community-involved initiatives, to inform interventions are critical to designing effective programs that recognize the heightened vulnerability to firearm violence exposure experienced by American Indian or Alaska Native and Black populations as a phenomenon resulting from cyclical systemic inequities.
